# Serum Interleukin-8 Levels and Their Association with Anxiety and Functional Disability in Military Personnel with Chronic Low Back Pain

**DOI:** 10.3390/jcm14113761

**Published:** 2025-05-27

**Authors:** Rim Dhahri, Hiba Ben Ayed, Ismail Dergaa, Halil İbrahim Ceylan, Aymen Tazaghdanti, Radhia Kochkar, Ezzedine Ghazouani, Insaf Fenniche, Lobna Ben Ammar, Refka Jebri, Imen Dorgham, Maroua Slouma, Raul-Ioan Muntean, Imen Gharsallah

**Affiliations:** 1 Rheumatology Department, Military Hospital of Instruction of Tunis, Tunis 1006, Tunisia; rimdhahri@ymail.com (R.D.); benayedhiba3@gmail.com (H.B.A.); insaffenniche@gmail.com (I.F.); lobna.b.ammar@gmail.com (L.B.A.); refkajebri6@gmail.com (R.J.); dorgam.imen@fmt.utm.tn (I.D.); maroua.slouma@gmail.com (M.S.); imengharsallah2005@gmail.com (I.G.); 2 High Institute of Sport and Physical Education of Ksar Said, University of Manouba, Manouba 2010, Tunisia; phd.dergaa@gmail.com; 3 High Institute of Sport and Physical Education of El Kef, University of Jendouba, El Kef 7100, Tunisia; 4 Physical Education of Sports Teaching Department, Faculty of Sports Sciences, Atatürk University, Erzurum 25240, Türkiye; 5 Immunology Department, Military Hospital of Instruction of Tunis, Tunis 1006, Tunisia; tez.aymen@fmt.utm.tn (A.T.); radhiakochkar@yahoo.fr (R.K.); ghazezz@yahoo.fr (E.G.); 6 Department of Physical Education and Sport, Faculty of Law and Social Sciences, University “1 Decembrie 1918” of Alba Iulia, 510009 Alba Iulia, Romania

**Keywords:** biomarkers, immunoassay, inflammation, nociception, psychoneuroimmunology, radiography, rehabilitation, spine

## Abstract

**Background/Objectives:** Chronic low back pain (LBP) remains a leading cause of disability and healthcare utilization globally, with complex, multifactorial pathophysiology. Despite advances in imaging, diagnosis often remains challenging due to poor correlation between structural findings and clinical symptoms. Recent evidence suggests inflammatory mechanisms may underlie persistent pain. This study investigated whether systemic inflammatory cytokines are altered in military personnel with chronic LBP and examined their relationships with clinical manifestations, psychological factors, and radiological findings. **Methods:** In this cross-sectional study, we enrolled 50 patients with chronic non-specific LBP (duration ≥ 3 months) and 50 age-, sex-, and BMI-matched healthy controls. All patients underwent a comprehensive clinical assessment, which included evaluation of pain intensity (VAS), neuropathic pain screening (DN4), psychological assessment (HADS), fibromyalgia screening (FIRST), and assessment of functional disability (Oswestry Disability Index and Roland-Morris Disability Questionnaire, EIFEL). Serum levels of IL-6, IL-8, IL-1β, TNF-α, and IL-10 were measured using chemiluminescence and enzyme-linked immunosorbent assay (ELISA) techniques. Radiological findings were documented through MRI and CT imaging of the lumbar spine. **Results:** Serum IL-8 levels were significantly elevated in patients with chronic LBP compared to healthy controls (8.52 ± 6.7 vs. 4.8 ± 0.56 pg/mL, *p* < 0.001). Weak positive correlations were observed between IL-8 levels and anxiety scores (r = 0.3, *p* = 0.02) and functional disability, as measured by the EIFEL questionnaire (r = 0.3, *p* = 0.04); however, these associations did not remain significant after Bonferroni correction for multiple testing. Similarly, IL-6 showed a weak positive correlation with BMI (r = 0.21, *p* = 0.03) and a weak negative correlation with lumbar mobility, as assessed by Schober’s test (r = −0.38, *p* = 0.03), which also did not survive correction for multiple comparisons. **Conclusions:** This study identified serum IL-8 as a potential biomarker for chronic LBP. While we observed associations between specific inflammatory markers and psychological distress and functional disability, these correlations were weak and did not remain significant after correction for multiple testing. These preliminary findings suggest possible connections between inflammation and the psychophysiological aspects of chronic LBP that warrant further investigation in larger cohorts.

## 1. Introduction

Low back pain (LBP) represents a global health challenge of epidemic proportions. It ranks as the foremost cause of disability worldwide, with a point prevalence of 7.5% and a lifetime prevalence exceeding 80% [1,2]. The socioeconomic burden is substantial, accounting for 4% of emergency department attendances [3] and 15% of workplace absenteeism [4]. Furthermore, LBP stands as the leading chronic health condition forcing older workers into premature retirement [5].

LBP encompasses a heterogeneous set of conditions rather than a singular disease entity [2]. Non-specific LBP, defined by the absence of identifiable pathoanatomic causes such as trauma, infection, compression fracture, or malignancy, constitutes the most prevalent form [1,2,4]. This classification typically encompasses degenerative disc disease, lumbar disc herniation, facet joint osteoarthritis, and spinal stenosis [1,2]. The pathophysiology of LBP encompasses multiple pain mechanisms, including nociceptive, neuropathic, and occasionally nociplastic components that often overlap [1]. Neuropathic pain predominantly manifests in lumbar radiculopathy, characterized by pain, numbness, and/or weakness that radiates along the sciatic nerve from the lower back to the leg [6].

The complex nature of LBP partially explains the poor correlation between observed pathology and reported symptoms. Although traditionally conceptualized as a primarily mechanical injury to nerve roots, growing evidence suggests that inflammatory processes may underlie the development and persistence of radicular pain [4,7,8,9]. Intervertebral disc degeneration, widely recognized as a pivotal factor in LBP and radiculopathy, appears to be strongly correlated with the aberrant production of pro-inflammatory cytokines [4,8,10]. Key inflammatory mediators implicated in this process include tumor necrosis factor-alpha (TNF-α), interleukin-1β (IL-1β), interleukin-6 (IL-6), and interleukin-8 (IL-8), which collectively trigger pathogenic cascades within intervertebral disc cells [4,8,10]. Supporting this inflammatory hypothesis, studies have demonstrated elevated serum levels of TNF-α in approximately half of patients with severe back pain compared to only 15% of healthy controls [11]. Concurrently, anti-inflammatory cytokines such as interleukin-10 (IL-10) may inhibit pro-inflammatory responses, potentially representing an endogenous analgesic system [10,12].

Despite these advances, significant knowledge gaps persist regarding the relationship between systemic inflammatory markers and clinical manifestations of LBP. While local inflammation within degenerating discs has been well-documented, less is known about whether these inflammatory signals manifest systemically and how they correlate with specific pain characteristics, psychological factors, and functional outcomes. Most studies have focused predominantly on TNF-α and IL-6, with less attention given to other potentially essential cytokines such as IL-8. Furthermore, the relationship between serum cytokine levels and radiological findings remains poorly understood, as does their association with psychological comorbidities frequently observed in chronic pain populations.

Given these knowledge gaps, we aimed to assess whether pro- and anti-inflammatory cytokines could be detected in the serum of patients with chronic LBP compared with healthy subjects, and whether their levels correlate with subjective pain ratings, psychological factors, functional disability, and radiological findings.

## 2. Materials and Methods

We conducted a cross-sectional study from 1 January to 31 March 2020, in the rheumatology department at the Military Hospital of Tunis, Tunisia. The study was conducted during the early phase of the COVID-19 pandemic. We implemented comprehensive safety protocols to protect all participants and research staff from potential infection. All study procedures adhered to the institutional COVID-19 guidelines established by the Military Hospital of Tunis. All participants underwent health screenings before each clinical assessment session, which included temperature checks and symptom questionnaires. Physical distancing was maintained whenever possible, and all examination rooms were disinfected between participants according to hospital infection control protocols. Personal protective equipment was worn by clinical staff during all participant interactions, and hand hygiene was strictly enforced. These precautionary measures were implemented in accordance with national health authority recommendations and did not compromise the integrity of the data collection procedures [13,14].

### 2.1. Ethical Approval

This study was conducted in accordance with the Declaration of Helsinki and was approved by the Ethics Committee of the Military Hospital of Tunis (Decision No. 28/2019). All participants provided written informed consent before enrolment after receiving a detailed explanation of the study procedures, potential risks, and benefits. Confidentiality and anonymity of personal data were maintained throughout the study process.

### 2.2. Sample Size Calculation

Sample size was calculated based on the findings of Wang et al. [9], who reported a significant difference in serum IL-8 levels between patients with lumbar radicular pain and healthy controls. Using their reported mean IL-8 values of 7.32 pg/mL (SD = 5.21) in patients with lumbar radicular pain and 3.06 pg/mL (SD = 2.25) in healthy controls, we calculated the standardized effect size (Cohen’s d) as 1.06. With this effect size, a two-sided significance level of 0.05, and a desired power of 90%, the required sample size was determined to be 41 subjects per group using the formula:

n = 2[(zα/2 + zβ)^2^σ^2^]/Δ^2^

where:

zα/2 is the critical value of the normal distribution at α/2 (for a confidence level of 95%, α is 0.05, and zα/2 is 1.96).

zβ is the critical value of the normal distribution at β (for a power of 90%, β is 0.10, and zβ is 1.28).

σ^2^ is the pooled variance of the two groups.

Δ is the difference in means.

We enrolled 50 subjects per group to account for potential dropouts and to increase the statistical power for secondary analyses. The sample size calculation was double-checked using ChatGPT-4, following Methenni et al.’s [15] guidelines, and the same result was obtained.

### 2.3. Study Design and Participants

A total of 50 patients with non-specific LBP and/or sciatica were recruited from the outpatient clinic of our rheumatology department. The inclusion criteria were age between 20 and 55 years, pain lasting for at least 3 months, patients who had a computed tomography (CT) or a magnetic resonance imaging (MRI) scan of the lumbar spine in the 12 months preceding the study and who did not have an analgesic drug in the preceding 24 h. Exclusion criteria were body mass index (BMI) > 35 kg/m^2^, LBP due to infectious spondylodiscitis, spondylarthritis, vertebral fracture or metastasis, cauda equina syndrome, history of neoplasm, asthma, infections, chronic bowel disease, psoriasis, inflammatory rheumatic disease, diabetic polyneuropathy, psychiatric disease, neurologic disorder, use of oral or systemic corticosteroid therapy in the preceding 3 months, previous lumbar spine surgery, generalized musculoskeletal pain, and pregnancy.

For the cytokine assay, and given the absence of standardized reference values for serum cytokine levels in our population, we recruited 50 healthy volunteers from the hospital’s sampling room. They were matched for age, sex, and BMI with the patients, had no history of low back pain, and met the study’s exclusion criteria. Additionally, they experienced no pain on the day of blood sampling.

### 2.4. Clinical Assessment

Demographic and clinical data, including age, gender, disease onset, radicular pain, and therapeutic management, were collected by the same rheumatologist for all patients. Nociceptive pain intensity was screened on a Visual Analog Scale (VAS) ranging from 0 to 10; in this scale, zero corresponds to “no pain” and 10 to “agonizing pain”. Patients were divided into three groups based on VAS values, using the cut-off points reported by Boonstra et al. in patients with chronic musculoskeletal pain [16]. Scores ≤ 3.4 correspond to mild pain, scores of [3.5–7.4] to moderate pain, and scores ≥ 7.5 to severe pain.

Neuropathic pain was assessed using the validated Arabic version of the “Douleur Neuropathique 4” (DN4) questionnaire [17], a clinician-administered questionnaire composed of four questions and ten items. A score of 1 is given to each positive item and a score of 0 to each negative item. The total score is calculated as the sum of the ten items and the cut-off value for diagnosing neuropathic pain is a total score of 4/10 [17].

The functional and professional impact of pain was assessed based on the number of days of work absenteeism caused by LBP. The Arabic Hospital Anxiety Depression Scale (HADS) was used to assess depression and anxiety [18]. It comprises 14 items (7 items each for anxiety and depression), with a score ranging between 0 and 21 for the anxiety and depression subscales. Scores between 8 and 10 indicate moderate symptoms, whereas a score greater than 11 indicates significant symptoms that likely correspond with a clinical diagnosis [18].

Patients were screened for fibromyalgia using the validated Arabic version of the Fibromyalgia Rapid Screening Tool (FiRST) [19]. This self-administered questionnaire consists of six questions covering various dimensions of fibromyalgia, including widespread pain, fatigue, pain characteristics, non-painful abnormal sensations, functional somatic symptoms, sleep disturbances, and cognitive problems. It requires yes-or-no responses [19]. Each item corresponds to one point and a cut-off score of 5–6 is considered positive for FM [19].

The functional status of the patients was assessed using the Arabic version of the Oswestry Disability Index (ODI) [20], a self-administered questionnaire divided into ten sections designed to consider limitations of various activities of daily living. Each section is scored on a 0–5 scale, with 5 representing the most significant disability. The index is calculated by dividing the summed score by the total possible score, which is then multiplied by 100 and expressed as a percentage. Thus, the denominator is reduced by 5 for every question not answered. If a patient marks multiple statements in a question, the highest-scoring statement is recorded as an accurate indication of disability [20]. We also utilized the French version of the Roland–Morris Disability Questionnaire (EIFEL disability questionnaire) [21], which consists of 24 items describing behaviors related to activities of daily living. It is scored by adding up the number of selected items, with a total score ranging from 0 (no disability) to 24 (severe disability) [21].

All patients underwent a BMI and body composition assessment using an impedance meter, including muscle mass, fat mass, and body water [22]. Lumbar spine mobility was evaluated using Schober’s test [23]. A restricted lumbar motion was defined as a difference between the measurements in erect and flexion positions of less than 5 cm [23]. The presence of a discoradicular conflict was assessed using the crossed straight leg raising test (Lasegue’s sign), Leri’s sign, and the doorbell sign [24].

### 2.5. Radiological Assessment

All plain lumbar spine radiographs, CT scans, and MRIs were interpreted by a trained radiologist blinded to the clinical findings.

### 2.6. Cytokine Measurement

Venous blood was collected between 9:00 AM and 12:00 PM to minimize variability resulting from diurnal variations. Blood samples were drawn into dry glass tubes, centrifuged at 1500× *g* for 15 min, and stored at −80 °C until analysis.

IL-6, IL-8, IL-1β, and TNF-α levels were measured using the chemiluminescence method (IMMULITE 1000^®^, Siemens Healthcare Diagnostics, Erlangen, Germany). In contrast, IL-10 levels were measured using the enzyme-linked immunosorbent assay (ELISA) with the Human IL-10 ELISA Kit (Thermo Fisher Scientific, Waltham, MA, USA) according to the manufacturer’s protocol.

### 2.7. Statistical Analysis

All statistical analyses were performed using IBM SPSS Statistics version 25.0 (IBM Corp., Armonk, NY, USA). Quantitative variables were expressed as the mean ± standard deviation (SD) and the minimum and maximum values. Categorical variables were presented as frequencies and percentages. The normality of continuous data was assessed using the Shapiro–Wilk test. For comparisons between patients and healthy controls, the independent samples *t*-test was used for normally distributed variables, and the Mann–Whitney U test was applied for non-normally distributed variables. The Chi-square test was used to compare categorical data; where assumptions were violated, Fisher’s exact test was employed. Correlations between cytokine levels and clinical or demographic parameters were analyzed using Pearson’s correlation coefficient for normally distributed variables and Spearman’s rank correlation coefficient for non-normally distributed variables. The strength of correlation coefficients was interpreted according to standard thresholds: r = 0.00–0.19 (very weak), 0.20–0.39 (weak), 0.40–0.59 (moderate), 0.60–0.79 (strong), and 0.80–1.00 (very strong) [25]. Bonferroni correction was applied to adjust for the risk of type I error due to multiple comparisons, resulting in an adjusted significance threshold of α′ = 0.05/32 = 0.00156. All tests were two-tailed, and statistical significance was defined as *p* ≤ 0.05 unless otherwise stated.

## 3. Results

### 3.1. Epidemiological and Clinical Characteristics of Patients

The study included 50 patients with chronic LBP. The mean age was 41.9 ± 8.4 years, and the sex ratio (M:F) was 4.5:1. All patients were military personnel, 80% being field operatives. The mean LBP duration was 66.4 ± 12.9 months. All patients had chronic LBP and 78% had associated sciatica, which was bilateral in 28.2% of cases.

The mean VAS for back pain was 4.5 ± 1.9, with severe pain (VAS ≥ 7.5) reported in 24% of patients. The mean VAS for radicular pain was 2.6 ± 2.5, with severe radicular pain observed in 10% of patients. Neuropathic pain (DN4 ≥ 4) was identified in 26% of patients. The mean ODI and EIFEL disability scores were 44.6 ± 23.4% and 11.3 ± 5.7, respectively. Fibromyalgia (FiRST ≥ 5) was diagnosed in 12% of participants, while anxiety (HADS anxiety ≥ 11) and depression (HADS depression ≥ 11) were present in 38% and 36% of patients, respectively.

The mean BMI was 27 ± 3.7 kg/m^2^. Spine mobility limitation was observed in 30% of patients, with a mean Schober’s test index of +3.8 ± 1.2 cm. The straight-leg raising test was positive in 18% of patients. The doorbell sign was positive in 28% of patients, and the Leri sign in 8%. The detailed clinical and epidemiological characteristics of patients are summarized in Table 1.

### 3.2. Radiological Findings

Lumbar spine plain radiographs were normal in 40% of patients. CT examination was performed in 80% of patients (n = 40), revealing disc protrusion as the most frequent finding (82.5%). MRI was performed in 22 patients (44%) and demonstrated disc protrusion in 77% of cases, disc herniation in 27.3%, and discoradicular conflict in 46%. Modic type 1 and Modic type 2 vertebral endplate changes were observed in 22.7% and 4.3% of patients, respectively.

### 3.3. Cytokine Serum Levels

Serum IL-8 was significantly higher in patients with LBP than healthy controls (8.52 ± 6.7 vs. 4.8 ± 0.56 pg/mL, *p* < 0.001). There were no significant differences between the two groups regarding the serum levels of IL-6 (2.41 ± 0.82 vs. 2.66 ± 0.73 pg/mL, *p* = 0.06), IL-10 (0.13 ± 0.47 vs. 0.09 ± 1.7 pg/mL, *p* = 0.11), or TNF-α (7.31 ± 2.83 vs. 7.19 ± 1.6 pg/mL, *p* = 0.55). IL-1β was undetectable (<5 pg/mL) in both patients and controls. The cytokine serum levels in both groups are presented in Table 2.

### 3.4. Comparison of Inflammatory Cytokine Levels Based on Clinical Parameters

No significant differences in cytokine serum levels were observed among patients when stratified by gender, presence of radicular pain, neuropathic pain characteristics, pain intensity, or fibromyalgia status. For instance, IL-8 levels were similar in patients with radicular pain (8.49 ± 6.54 pg/mL) and those without (8.52 ± 6.86 pg/mL, *p* = 0.69). Similarly, IL-8 levels did not differ significantly among patients with mild (7.68 ± 5.8 pg/mL), moderate (9.19 ± 7.7 pg/mL), or severe (7.3 ± 3.11 pg/mL) back pain (*p* = 0.56). The detailed comparisons of cytokine levels based on clinical parameters are summarized in Table 3.

### 3.5. Comparison of Inflammatory Cytokine Levels Based on Radiological Parameters

No significant differences in cytokine serum levels were found among patients when analyzed according to different MRI findings, including disc herniation, Modic changes, or discoradicular conflict. IL-8 levels were comparable between patients with disc herniation (8.41 ± 5.15 pg/mL) and those without (8.6 ± 7.12 pg/mL, *p* = 0.71). Similarly, IL-8 levels did not significantly differ between patients with (10.1 ± 8.48 pg/mL) and without (7.09 ± 4.45 pg/mL) discoradicular conflict (*p* = 0.21). These comparisons are presented in Table 4.

### 3.6. Correlations Between Clinical Parameters and Cytokine Serum Levels

Weak positive correlations were identified between IL-8 levels and anxiety (assessed by the HADS) (r = 0.3, *p* = 0.02) and EIFEL functional disability score (r = 0.3, *p* = 0.04). IL-6 was weakly positively correlated with BMI (r = 0.21, *p* = 0.03) and weakly negatively correlated with Schober’s test measurements (r = −0.38, *p* = 0.03). After applying Bonferroni correction for multiple comparisons (approximately 32 tests, adjusted significance threshold *p* < 0.00156), none of these correlations remained statistically significant. No significant correlations were found between serum levels of IL-6, IL-8, IL-10, TNF-α, and pain intensity (VAS), neuropathic pain (DN4), fibromyalgia (FIRST), depression (HADS), or various radiological findings.

## 4. Discussion

Our study aimed to assess whether pro- and anti-inflammatory cytokines could be detected in the serum of patients with chronic LBP compared to healthy subjects, and whether their levels correlate with clinical manifestations, psychological factors, and radiological findings. The primary finding was that serum IL-8 levels were significantly higher in patients with chronic LBP compared to healthy controls. Additionally, we found significant positive correlations between IL-8 levels and both anxiety and functional disability, as well as between IL-6 and BMI. At the same time, IL-6 was negatively correlated with lumbar mobility assessed by Schober’s test. Importantly, no correlations were observed between cytokine levels and pain intensity, neuropathic characteristics, or radiological findings.

### 4.1. Elevated IL-8 Levels in Patients with Chronic LBP

Our study demonstrated significantly higher serum IL-8 levels in patients with chronic LBP than in healthy controls (*p* < 0.001). This finding aligns with results from Wang et al. [9] and Slouma et al. [8]. In the latter study, IL-8 was found to differentiate patients with LBP from pain-free controls with a cutoff of 4.5 pg/mL, demonstrating a sensitivity of 91.3% and a specificity of 90% [8]. Similarly, Krock et al. [26] observed elevated IL-8 levels in the cerebrospinal fluid of patients with chronic LBP compared to pain-free subjects with or without disc degeneration, suggesting that IL-8 elevation is specific to symptomatic patients rather than merely reflecting structural degeneration.

The source of increased IL-8 production in LBP patients remains incompletely understood. Some investigators have proposed neuroinflammatory mechanisms involving the dorsal root ganglia, the spinal cord, or supraspinal structures [27,28]. According to this hypothesis, nerve injury may compromise the blood-spinal cord barrier, facilitating transport of peripheral cytokines into the cerebrospinal fluid and subsequently to the degenerated disc. Alternatively, others suggest that painful degenerating discs may produce IL-8 locally, with subsequent leakage into the cerebrospinal fluid [26]. This is supported by Ahn et al. [29], who found that increased IL-8 mRNA levels in herniated discs were associated with radicular pain during back extension. Further supporting the pathogenic role of IL-8, Krock et al. [26] demonstrated that chronic inhibition of chemokine receptor type 1, a receptor for IL-8, reduced behavioral signs of back pain and decreased disc inflammation in an animal model.

### 4.2. Correlation Between IL-8 and Anxiety

Our analysis revealed a significant positive correlation between serum IL-8 levels and anxiety as assessed by the HADS (r = 0.3, *p* = 0.02). While this association differs from the findings of Slouma et al. [8], who reported a correlation between IL-8 and pain intensity rather than psychological factors, the relationship between IL-8 and anxiety is well-documented in psychiatric literature [30,31]. Another study conducted by Tang et al. [32] demonstrated significantly higher serum IL-8 levels in patients with first-episode generalized anxiety disorder (GAD) compared to healthy controls, with a significant positive correlation between anxiety measures and IL-8 levels. Furthermore, Hou et al. [33] showed that selective serotonin reuptake inhibitor treatment for 12 weeks significantly reduced both baseline anxiety levels and pro-inflammatory cytokines, including IL-8, in patients with GAD. However, it should be noted that the correlation we observed was weak (r = 0.3) and did not remain significant after correction for multiple testing, suggesting that this relationship should be interpreted with caution and verified in larger studies.

This association between IL-8 and anxiety in our LBP population suggests a potential psychoneuroimmunological mechanism that may contribute to the psychological burden of chronic pain. The bidirectional relationship between inflammatory processes and psychological distress could represent a target for therapeutic interventions that address both the physical and psychological dimensions of chronic LBP.

### 4.3. Correlation Between IL-8 and Functional Disability

Our study identified IL-8 as the only pro-inflammatory cytokine positively correlated with functional disability, assessed by the EIFEL score (r = 0.3, *p* = 0.04). This finding contrasts with the results from other studies [9,11,34]. Wang et al. [9] found that disability (assessed by ODI) significantly correlated with IL-6 (r = 0.394, *p* = 0.013) and TNF-α (r = 0.629, *p* = 0.001), but not with IL-8 (r = −0.133, *p* = 0.418). Similarly, Aripaka et al. [34] reported significant positive correlations between ODI and IL-6 expression in annulus fibrosus and nucleus pulposus, while TNF-α correlated only with pain intensity. It is essential to acknowledge that the correlation we observed (r = 0.3) was weak and did not withstand the Bonferroni correction for multiple testing, thereby limiting the strength of our conclusions regarding this relationship.

These disparate findings may reflect the complex and potentially overlapping pathways through which different inflammatory cytokines influence functional outcomes. As noted by Aripaka et al. [34], pain and disability may not be strongly linked biochemically, suggesting distinct but overlapping mechanisms. Our findings suggest that IL-8 may play a more significant role in functional limitation than previously recognized, potentially through its impact on neuromuscular function, psychological state, or central pain processing.

### 4.4. Correlation Between IL-6, BMI, and Lumbar Mobility

We observed a significant positive correlation between IL-6 levels and BMI (r = 0.21, *p* = 0.03), consistent with established knowledge regarding adipose tissue function. Hypertrophied adipose tissue, now recognized as an endocrine organ, produces increased pro-inflammatory cytokines, particularly IL-6 [35]. Obese individuals typically present with elevated C-reactive protein (CRP) levels, primarily produced in the liver in response to IL-6 [35]. Some evidence suggests that CRP itself may participate in signaling pathways responsible for musculoskeletal pain sensation and activation [36], potentially representing another mechanism through which obesity exacerbates LBP.

Additionally, we found that IL-6 was negatively correlated with lumbar mobility as assessed by Schober’s test (r = −0.38, *p* = 0.03). While few studies have examined the relationship between inflammatory markers and spine mobility, Slouma et al. [8] reported significantly higher IL-17 levels in LBP patients with spine limitations. Recent research by Canseco et al. [37] offers a potential mechanistic explanation for this association. Their study demonstrated improved back mobility in 17.5% of patients with comorbid degenerative spinal disease and migraine following treatment with anti-calcitonin gene-related peptide (CGRP). The authors attributed this improvement to CGRP inhibition, as this peptide upregulates pro-inflammatory mediators, including TNF-α, IL-6, brain-derived neurotrophic factor, and nerve growth factor in intervertebral disc cells [37]. Our finding of a negative correlation between IL-6 and Schober’s test suggests that IL-6-mediated inflammation may contribute to reduced spinal mobility in chronic LBP patients. Both correlations (r = 0.21 for BMI and r = −0.38 for Schober’s test) were weak and did not remain significant after appropriate correction for multiple testing, indicating that these relationships should be considered preliminary findings that require further validation.

### 4.5. Lack of Correlation Between Cytokines and Pain Intensity or Radiological Findings

Contrary to our expectations and some previous reports [4,8], we found no significant correlations between serum levels of IL-6, IL-8, IL-10, or TNF-α and pain intensity (VAS), neuropathic pain characteristics (DN4), or radiological findings. This absence of correlation between inflammatory markers and clinical and imaging parameters highlights chronic LBP’s complex and multifactorial nature.

The disconnect between inflammatory markers and pain intensity may reflect the involvement of multiple pain mechanisms beyond inflammation, including neuropathic, nociceptive, and nociplastic components [1]. Similarly, the lack of correlation with radiological findings aligns with the well-established clinical observation that structural abnormalities on imaging studies often correlate poorly with symptoms [1,2].

### 4.6. Strengths and Limitations

The primary strength of our study lies in its comprehensive assessment of multiple inflammatory cytokines (IL-6, IL-8, IL-10, TNF-α, and IL-1β) in patients with chronic LBP, accompanied by detailed clinical, psychological, and radiological evaluations. This approach allowed us to examine the relationships between inflammatory markers and various dimensions of the LBP experience, including physical, functional, and psychological aspects. However, we acknowledge several limitations. First, our small sample size may limit the statistical power to detect weaker associations. Second, our study population consisted exclusively of military personnel, which may limit the generalizability of our findings to broader LBP populations. Third, several factors influencing cytokine concentrations, such as physical activity levels and dietary patterns, were not assessed. Fourth, we measured cytokines in serum rather than in cerebrospinal fluid or local disc tissue, which may more accurately reflect the inflammatory environment at the site of pathology. Fifth, although we employed validated questionnaires to assess pain intensity, neuropathic features, and fibromyalgia screening, the lack of more in-depth mechanistic pain profiling, such as specific differentiation between nociceptive, neuropathic, and nociplastic pain, may limit the interpretation of the underlying pain mechanisms. Finally, the cross-sectional design precludes the establishment of causal relationships between cytokine levels and clinical parameters.

### 4.7. Clinical Implications and Future Directions

Our findings provide preliminary evidence of an association between chronic LBP and pro-inflammatory cytokines, particularly IL-8 and IL-6. While we identified potentially interesting correlations between these cytokines and clinical parameters, these associations were weak and did not withstand correction for multiple testing. Nevertheless, these preliminary observations suggest that further investigation of inflammatory pathways might yield insights into potential therapeutic approaches for pain management in patients with degenerative disc disease. The observed relationships between IL-8 and both anxiety and functional disability, though weak, highlight the importance of addressing psychological factors in LBP management and suggest potential benefits from integrated interventions targeting both inflammation and psychological distress.

However, the high cost of cytokine inhibitors and the high prevalence of LBP, which typically follows a benign course in most patients, may limit the feasibility of such approaches, particularly in resource-constrained settings. Future research should focus on identifying specific LBP subgroups most likely to benefit from anti-inflammatory interventions, developing more accessible anti-inflammatory approaches, and exploring the longitudinal relationships between inflammatory markers and clinical outcomes in chronic LBP.

## 5. Conclusions

Our study identified serum IL-8 as a potential biomarker for chronic LBP. While we observed associations between IL-8 and psychological distress and functional disability, these correlations were weak and did not remain significant after correction for multiple testing. These preliminary findings suggest that IL-8-mediated inflammation may contribute to the psychophysiological aspects of chronic LBP. Still, larger, adequately powered studies are needed to confirm these relationships and further explore potential therapeutic targets addressing the complex biopsychosocial nature of this condition.

## Figures and Tables

**Table 1:** Demographic and clinical characteristics of patients.

Characteristics	Patients (n = 50)	Pain-Free Controls (50)
Age (years) (mean ± SD)	42 ± 8.4 years	44 ± 7.2 years (*p* = 0.97)
Men, n (%)	41 (82)	39 (78)
Comorbidities, n (%)	Hypertension: 2 (4)	0
	Diabetes: 4 (8)	0
Profession, n (%)	Active military personnel: 40 (80)	
	Administrative staff: 9 (20)	
	Retired: 1	
LBP duration (months) (mean ± SD)	66.4 ± 12.9	
Radicular pain, n (%)	39 (78)	0
VAS back pain (mean ± SD)	4.5 ± 1.9	0
VAS radicular pain (mean ± SD)	2.6 ± 2.5	0
Neuropathic pain, n (%)	13 (26)	0
LBP-related work absenteeism (days), (mean ± SD)	21 ± 3.3	
Physiotherapy interventions, n (%)	24 (48)	
Mood disorder according to the HADS, n (%)	Anxiety: 19 (38)	
	Depression: 18 (36)	
Fibromyalgia (FiRST ≥ 5), n (%)	6 (12)	
ODI, (mean ± SD) %	44.6 ± 23.4	
EIFFEL disability score (mean ± SD)	11.3 ± 5.7	
BMI (mean ± SD) kg/m^2^	27 ± 3.7	27.9 ± 4.1 (*p* = 0.23)
Body composition, (mean ± SD) %	Fat mass: 28.2 ± 8.3	29.2 ± 8.4 (*p* = 0.77)
	Muscle mass: 40.2 ± 8.1	37.1 ± 5.4 (*p* = 0.16)
	Body water: 52 ± 6.1	51.8 ± 6.7 (*p* = 0.86)
Schober’s index, (mean ± SD) cm	+3.8 ± 1.2	
Spine limitation, n (%)	15 (30)	
Positive disco-radicular conflict signs, n (%)	Doorbell sign: 14 (28)	
	Lasegue’s sign: 9 (18)	
	Leri sign: 4 (8)	

LBP: low back pain, VAS: Visual Analog Scale, HADS: the Hospital Anxiety Depression Scale, FiRST: Fibromyalgia Rapid Screening Tool, ODI: the Oswestry Disability Index, EIFFEL disability score: the French version of the Roland–Morris Disability index, BMI: body mass index.

**Table 2:** Comparison of inflammatory cytokine serum levels between patients and healthy controls.

	Patients (50)	Pain-Free Controls (50)	*p*
IL-6	2.41 ± 0.82	2.66 ± 0.73	0.06
IL-8	8.52 ± 6.7	4.8 ± 0.56	0.001
IL-10	0.13 ± 0.47	0.09 ± 1.7	0.11
TNF-α	7.31 ± 2.83	7.19 ± 1.6	0.55
IL-1β	<5 *	<5	

Values were expressed as mean ± SD, IL: interleukin, TNF: tumor necrosis factor. *: Below detection threshold.

**Table 3:** Comparison of inflammatory cytokine serum levels based on clinical parameters.

	Radicular Pain		*p*	VAS Back Pain			*p*	Neuropathic Pain		*p*
	Yes (n = 39)	No (n = 11)		Mild (n = 15)	Moderate (n = 29)	Severe (n = 6)		Yes (n = 13)	No (n = 37)	
IL-6	2.2 ± 1.17	2.27 ± 0.8	0.9	2.01 ± 0.46	2.32 ± 0.75	2.57 ± 1.36	0.24	1.89 ± 1.1	2.2 ± 1.3	0.15
IL-8	8.49 ± 6.54	8.52 ± 6.86	0.69	7.68 ± 5.8	9.19 ± 7.7	7.3 ± 3.11	0.56	8.12 ± 5.7	7.13 ± 2.8	0.31
IL-10	0.09 ± 0.17	0.16 ± 0.53	0.21	0.2 ± 0.64	0.12 ± 0.41	0.21 ± 0.09	0.65	0.19 ± 0.12	0.15 ± 0.08	0.81
TNF-α	6.9 ± 1.26	7.42 ± 2.6	0.85	6.85 ± 1.55	7.43 ± 1.12	7.86 ± 2.12	0.4	6.87 ± 1.3	7.3 ± 1	0.24

IL: interleukin, TNF: tumor necrosis factor, VAS: Visual Analog Scale, *p*: probability value.

**Table 4:** Comparison of inflammatory cytokine serum levels based on radiological findings.

	Disc Herniation		*p*	MODIC Changes			*p*	Discoradicular Conflict		*p*
	Yes (n = 12)	No (n = 38)		Absent (n = 16)	Type 1 (n = 5)	Type 2 (n = 2)		Yes (n = 23)	No (n = 27)	
IL-6	2.25 ± 0.37	2.27 ± 0.83	0.26	2 ± 0.36	3.37 ± 0.73	2 ± 1.1	0.82	2.37 ± 0.82	2.16 ± 0.66	0.1
IL-8	8.41 ± 5.15	8.6 ± 7.12	0.71	7.33 ± 4.18	8.2 ± 5.7	8.3 ± 3.1	0.16	10.1 ± 8.48	7.09 ± 4.45	0.21
IL-10	0.5 ± 1	0.06 ± 0.21	0.52	0.09 ± 0.11	0.12 ± 0.33	0.1 ± 0.09	0.71	0.26 ± 0.67	0.01 ± 0.09	0.11
TNF-α	7.16 ± 1.2	7.7 ± 2.83	0.72	6.21 ± 0.95	8.33 ± 1.2	7.23 ± 1.18	0.76	7.84 ± 2.93	6.85 ± 1.7	0.1

IL: interleukin, TNF: tumor necrosis factor, VAS: Visual Analog Scale.

## Data Availability

Underlying data not included in this article are available from the corresponding author upon reasonable request.
